# Editorial: Advances in chronic kidney disease diagnosis and therapy

**DOI:** 10.3389/fmed.2023.1209571

**Published:** 2023-05-11

**Authors:** Hamad Ali, Filippo Pinto e Vairo, Ali AlSahow, Mohamed Abu-Farha

**Affiliations:** ^1^Department of Medical Laboratory Sciences, Faculty of Allied Health Sciences, Health Sciences Center, Kuwait University, Jabriya, Kuwait; ^2^Research Division, Dasman Diabetes Institute, Dasman, Kuwait; ^3^Department of Clinical Genomics, Center for Individualized Medicine, and Division of Nephrology and Hypertension, Mayo Clinic, Rochester, MN, United States; ^4^Division of Nephrology, Al-Jahra Hospital, Ministry of Health, Al-Jahra, Kuwait

**Keywords:** chronic kidney disease, glomerulopathy, tyrosine kinase inhibitors (TKI), ferroptosis 1, Receptor for Advanced Glycation End-product (RAGE), granulomatosis with polyangiitis (GPA), triglyceride-glucose index

Chronic kidney disease (CKD) continues to be a major challenge for health care systems globally. The disease is a direct contributor to morbidity and mortality, and a risk factor for cardiovascular disease, a leading cause of death globally. Recent studies indicated that CKD-related deaths were among the largest rises of causes of death between 2010 and 2019 which could be attributed, in part, to the increased prevalence of CKD risk factors including obesity, hypertension and diabetes (1). There is an urgent need to develop more effective and targeted treatments for CKD that can prevent/slow disease progression and improve patient outcomes. Furthermore, the identification of sensitive and reliable biomarkers is essential to monitor disease progression accurately, assess the effectiveness of treatments, and identify patients who may benefit from early interventions. Our Research Topic aimed to explore recent advances in the field of CKD to improve our understanding of the disease, leading to better diagnosis, management, and treatment options for patients.

Zhu et al. explored the relationship between Triglyceride-glucose (TyG) index and the risk of CKD. In their longitudinal observational study on more than 2,000 hypertensive participants with a median follow up of 31 months, they found that individuals in the highest quartile of TyG index had a 1.63-fold higher hazard ratio for the presence of CKD compared to those in the lowest quartile. Furthermore, the relationship between TyG index and CKD was found to be non-linear, with a rapid increase in risk beyond a TyG index of 8.94. The study argued that early intervention of metabolic factors may prevent the occurrence of CKD and reduce the incidence of cardiovascular disease and premature death. There is a bi-directional relationship between CKD and hypertension, as the latter can serve as both a result and a risk factor in the development and progression of CKD. Prasad et al. investigated the renal and cardiovascular outcomes associated with administration of Angiotensin-converting enzyme inhibitors (ACEI) and angiotensin receptor blockers (ARB) which are the preferred antihypertensive drug class in patients with CKD. Their findings showed that utilization of ACEI/ARB correlated with a slower decline in eGFR among individuals with CKD stages 1–3. ACEI/ARBs administration also correlated with a reduced risk of renal outcomes and cardiovascular mortality.

The Receptor for Advanced Glycation End-products (RAGE) is activated by multiple ligands such as AGEs, HMGB1, and S100 proteins. Its activation through these ligands triggers multiple pathways that are involved in various diseases ranging from autoimmune diseases to cancers and infectious diseases as well as the progression of both CKD and COVID-19. Curran and Kopp reviewed studies that can link RAGE ligands with those diseases and highlighted the elevated mortality risk associated with COVID-19 patients with CKD. They also reported the role played by the RAGE pathway in renal cell injury and dysfunction. Based on these elaborate involvement of RAGE pathways in both CKD and COVID-19, the authors proposed that interventions to reduce RAGE and RAGE ligand plasma and tissue levels can provide a promising therapeutic target to delay or stop the progression of COVID-19-associated CKD and perhaps non-COVID-19-associated CKD. As a result, more research is needed to advance this concept and to investigate the clinical safety and efficacy of RAGE-targeted therapies.

To better characterize and to identify the time-dependent risk associated with granulomatosis with polyangiitis (GPA), a lethal primary systemic vasculitides disease characterized by granulomatous and necrotizing inflammation affecting the upper respiratory tract, lungs, and kidneys, Lin et al. analyzed retrospective data from a nationally representative database in Taiwan for individuals with GPA. The analysis included GPA patients without end-stage kidney disease (ESKD) as well as their sex, age, entry time, and comorbidities in a matched control group at a 1:4 ratio (142 GPA patients and 568 controls). Their findings showed that GPA patients had the highest mortality rate within the first 6 months to a year and experienced incidence of ESKD exclusively in the first 3 years of diagnosis. In conclusion, the authors highlighted the need for close monitoring of GPA patients within the early years of the disease to protect against ESKD and to improve survival rate. To investigate the potential benefits of remote patient management (RPM) in reducing all-cause mortality among patients with heart failure (HF) and renal impairment Naik et al. initiated a prospective, randomized, controlled multicenter trial. The trial included 1,538 patients with stable heart failure from Germany between 2013 and 2017. Patients were randomized to either RPM plus usual care or usual care alone. The primary endpoint was the percentage of lost unplanned days as a result of cardiovascular hospitalizations or death while the secondary outcome included hospitalizations, all-cause, and cardiovascular mortality. Overall, the study showed that RPM can be a valuable approach in reducing all-cause mortality in the high-risk population of patients with heart failure and CKD.

Yang et al. reviewed the role of autophagy-dependent ferroptosis in kidney diseases. Ferroptosis is a distinct type of cell death that is mainly caused by iron toxicity and lipid peroxidation. In this review the authors provided a comprehensive overview of the role of ferroptosis in kidney disease, highlighting the major role of autophagy-dependent ferroptosis in renal impairment. ADAMTS9 is a metalloproteinase that has been shown to play a role in various kidney diseases. Pathogenic variants within this gene have been shown to cause nephronophthisis-related ciliopathies (NPHP-RC). In this issue, Yu et al. intended to confirm whether ADAMTS9 dysfunction causes NPHP and glomerulopathy, utilizing *ADAMTS9* knockout kidney organoids and single-cell RNA sequencing highlighting the functional roles of *ADMATS9* in glomeruli and tubules. Their data showed that the podocyte clusters followed by the proximal tubules expressed the highest levels of *ADAMTS9*. *ADAMTS9* knockout resulted in the activation of multiple pathways in podocyte clusters, including the Wnt/PCP signaling pathway, which requires further analysis. Tyrosine kinase inhibitors (TKIs) are effective tumor therapeutic targets but are associated with significant side effects such as hypertension, diarrhea, bleeding and proteinuria. Fu et al. were interested in evaluating the therapeutic value of Liuwei Dihuang Pill (LDP), which is an ancient Chinese medicine formula. The authors showed *in vitro* that LDP mitigated TKI-related proteinuria through the attenuation of inflammatory injury of podocytes.

Although recent progress in CKD diagnosis and therapy offers hope for better management, more targeted treatments are urgently needed to prevent/slow disease progression and improve patient outcomes. Additionally, the development of sensitive diagnostic and prognostic markers is critical for early detection and effective management, potentially reducing morbidity and mortality related to CKD.

## Author contributions

All authors listed have made a substantial, direct, and intellectual contribution to the work and approved it for publication.
